# Generalization and discrimination of inhibitory avoidance differentially engage anterior and posterior retrosplenial subregions

**DOI:** 10.3389/fnbeh.2024.1327858

**Published:** 2024-01-17

**Authors:** Erisa Met Hoxha, Payton K. Robinson, Kaitlyn M. Greer, Sydney Trask

**Affiliations:** ^1^Purdue University Department of Psychological Sciences, West Lafayette, IN, United States; ^2^Purdue University Institute for Integrative Neuroscience, West Lafayette, IN, United States; ^3^Purdue University Center on Aging and the Life Course, West Lafayette, IN, United States

**Keywords:** inhibitory avoidance, generalization, discrimination, retrosplenial cortex, basolateral amygdala

## Abstract

**Introduction:**

In a variety of behavioral procedures animals will show selective fear responding in shock-associated contexts, but not in other contexts. However, several factors can lead to generalized fear behavior, where responding is no longer constrained to the conditioning context and will transfer to novel contexts.

**Methods:**

Here, we assessed memory generalization using an inhibitory avoidance paradigm to determine if generalized avoidance behavior engages the retrosplenial cortex (RSC). Male and female Long Evans rats received inhibitory avoidance training prior to testing in the same context or a shifted context in two distinct rooms; one room that had fluorescent lighting (Light) and one that had red LED lighting (Dark).

**Results:**

We found that animals tested in a light context maintained context-specificity; animals tested in the same context as training showed longer latencies to cross and animals tested in the shifted context showed shorter latencies to cross. However, animals tested in the dark generalized their avoidance behavior; animals tested in the same context and animals tested in the shifted context showed similarly-high latencies to cross. We next examined expression of the immediate early gene zif268 and perineuronal nets (PNNs) following testing and found that while activity in the basolateral amygdala corresponded with overall levels of avoidance behaviors, anterior RSC (aRSC) activity corresponded with learned avoidance generally, but posterior RSC (pRSC) activity seemed to correspond with generalized memory. PNN reduction in the RSC was associated with memory formation and retrieval, suggesting a role for PNNs in synaptic plasticity. Further, PNNs did not reduce in the RSC in animals who showed a generalized avoidance behavior, in line with their hypothesized role in memory consolidation.

**Discussion:**

These findings suggest that there is differential engagement of retrosplenial subregions along the rostrocaudal axis to generalization and discrimination.

## Introduction

Fear- and anxiety-based disorders are the most common class of mental health disorders with a lifetime prevalence of 28% in the United States (Kessler et al., 2005). These disorders are often characterized by excessive avoidance (Salters-Pedneault et al., 2004), where an individual or an animal learns to minimize contact with an aversive stimulus or situation following a fear-inducing event (Krypotos et al., 2015). While this is a generally adaptive process, this type of learning can become maladaptive when it generalizes to safe or neutral situations (i.e., those that have not been paired with an aversive event). A better understanding of behavioral patterns and neurobiological mechanisms underlying generalized avoidance behavior will aid in development of treatment aiming to reduce symptoms associated with anxiety disorders.

In typical avoidance paradigms, an animal first learns that a neutral stimulus (conditional stimulus, or CS; e.g., a specific cue or context) predicts an unconditional stimulus (UCS; e.g., a footshock). Later, the animal will avoid the CS that previously predicted shock by performing some action, like pressing a lever or moving to a new area of the chamber. However, in inhibitory avoidance one side of a two-compartment chamber is paired with a footshock. Following this learning, rats will avoid entering the shock-associated side of the chamber by staying on the side that was not paired with the shock. During testing, latency to cross from the nonshock-associated side to the shock-associated side of the chamber is measured as an index of learning. Some types of aversive learning are context-dependent. In a variety of behavioral procedures, including inhibitory avoidance, animals will show selective fear responding to contexts where a shock was delivered, but not to other contexts (Zhou and Riccio, 1996; Wiltgen and Silva, 2007; Bonanno et al., 2023). This behavioral decrement when tested in a novel context, but not in the training context, suggests that animals can discriminate between these environments and use that information to guide behavior.

However, several factors can lead to a generalized fear response where fear behavior is no longer constrained to the acquisition context. For example, while inhibitory avoidance behavior is initially context-dependent and can be reduced by changing surrounding environmental cues, after 2 weeks rats will generalize this fear response to a new context (Zhou and Riccio, 1996). This generalization is reflected by similarly-long latencies to cross to the shock-associated side of the chamber when behavior was tested in either the same context as training or a shifted context. This suggests that 14 days following training animals were no longer using contextual cues to inform their behavior, aligning with work in context fear conditioning showing that memories generalize to new environments over time (Poulos et al., 2016; Pollack et al., 2018). Another factor that can influence generalization is memory strength. For example, context fear conditioning with five footshocks resulted in increased freezing to a neutral context (i.e., generalization) relative to training with three footshocks (Ortiz et al., 2019). Although these studies have informed our understanding of both the behavioral and neurobiological mechanisms of fear generalization, they have also conflated variables such as memory strength (Ortiz et al., 2019) and age (Zhou and Riccio, 1996). This poses a potential confound as the generalized memory is either trained or tested in a way that is qualitatively different from the non-generalized or specific memory.

Recent work has identified a way to produce generalization of avoidance to a new context without manipulating either memory strength or age (Oleksiak et al., 2021). Here, rats were trained in a two-way signaled active avoidance task in either a brightly-lit room or a dark room. Animals trained in the brightly-lit room and tested in the dark room showed less context-dependency than animals that were trained in the dark room and tested in the brightly-lit room. This demonstrates asymmetrical generalization of an avoidance response based on environmental conditions and suggests that memory generalization and specificity can be studied without manipulating memory age or strength between groups.

Most of this work has focused primarily on the hippocampus because of its well-known role in context-dependency of behavior (Kim and Fanselow, 1992; Maren et al., 1997; Anagnostaras et al., 1999; Rudy et al., 2002; Quinn et al., 2005; Zelikowsky et al., 2012; Oleksiak et al., 2021). However, as a memory becomes less precise, it no longer depends on the hippocampus (Wiltgen and Silva, 2007; Ortiz et al., 2019). Instead, as hippocampal-dependency is reduced, memory becomes stabilized in cortical regions through a process called systems consolidation. Interestingly, this systems-level consolidation corresponds with reduced memory specificity and increased generalization (Jasnow et al., 2017; de Sousa et al., 2019), a finding sometimes referred to as the forgetting of stimulus attributes (Riccio and Joynes, 2007). Elevated levels of freezing to a context other than the training context indicate that a memory or behavior has become context-independent; this transition coincides with a shift from a reliance on the hippocampus to a dependency on cortical regions like the anterior cingulate cortex (ACC; Frankland et al., 2004; Teixeira et al., 2006; Restivo et al., 2009; Ortiz et al., 2019; for review see Frankland and Bontempi, 2005). This transfer is likely mediated or facilitated by activity in the retrosplenial cortex (RSC; de Sousa et al., 2019), which has a time-independent role in memory retrieval and receives inputs from both the dorsal hippocampus and the anterior cingulate cortex (Sugar et al., 2011; Trask et al., 2021c).

Recent work identifying the unique contributions of the anterior and posterior subregions of the RSC has contributed to our understanding of *how* information is integrated during memory acquisition. For example, optogenetic inhibition of the anterior RSC (aRSC) during acquisition reduced later freezing to a discrete conditional stimulus (CS), while the same inhibition of the posterior RSC (pRSC) reduced subsequent freezing to the context (Trask et al., 2021a), suggesting dissociable roles for these subregions in memory acquisition. Moreover, unlike the time-limited roles for the hippocampus (i.e., recent) and the ACC (i.e., remote), RSC activity is needed at both recent and remote time points with blocking NMDA receptors in the RSC disrupting the retrieval of both recent and remote memories (Corcoran et al., 2011). Together, these results suggest that the retrosplenial cortex has a broad role in both memory consolidation and memory retrieval, even following systems consolidation.

Retrosplenial activity may therefore mediate generalization through a role in systems consolidation (de Sousa et al., 2019; for review see Trask et al., 2021c). For example, inhibiting protein synthesis in the RSC after training affected inhibitory avoidance memory 7 days later, but not 2 days later (Katche et al., 2013), suggesting that the RSC is important for maintaining remote, less precise memory. Further, optogenetic stimulation of the subset of neurons in the RSC active during conditioning following training increases memory generalization and ACC-dependence (de Sousa et al., 2019), supporting a role for the RSC in systems consolidation.

While systems consolidation can occur over a period of days to weeks, cellular processes associated with consolidation likely only last a few hours (Rudy and Sutherland, 2008). Transcriptional regulation of genes, including immediate early genes like zif268 (also called EGR-1) are indicative of brain activity during learning. Zif268 has been linked to active memory processes during acquisition and retrieval (Hoffman et al., 2015; Trask et al., 2021a,b; Bonanno et al., 2023) and memory consolidation (Bozon et al., 2003). Perineuronal nets (PNNs), a component of the extracellular matrix, have been hypothesized to have a role in memory processes. While PNNs degrade during memory formation (corresponding with increased synaptic plasticity), they become stabilized through memory consolidation (Wang and Fawcett, 2012; Sorg et al., 2016).

In the present experiments, we applied a similar framework to that established in Oleksiak et al. (2021) to test how generalization of inhibitory avoidance behavior influences neural activity in the RSC. We hypothesized that memory acquisition would be accompanied by an increase in expression of zif268 and a decrease in quantity of PNNs. We also predicted that subregions of the RSC would be differentially involved in the retrieval of context-specific and generalized inhibitory avoidance memory. We expected overall levels of avoidance behavior would correspond with elevated zif268 expression in the aRSC, pRSC, and basolateral amygdala (BLA). We chose to examine neural activity in the BLA as zif286 expression in this region closely corresponds with degree of fear behavior (e.g., Hoffman et al., 2015; Bonanno et al., 2023). However, we predicted that animals exhibiting memory specificity would have a greater number of PNNs in the pRSC, corresponding with better contextual discrimination, and animals demonstrating generalization of an inhibitory avoidance memory would have a greater number of PNNs in the aRSC.

## Methods

### Subjects

Age-matched (3-month) male (*n* = 36) and female (*n* = 36) Long Evans rats purchased from Envigo (Indianapolis, IN) were used. The animal colony was maintained on a 12:12 light:dark cycle and behavioral experiments were conducted during the light cycle. Animals were acclimated to the colony for 7 days prior to experimentation. All groups consisted of equal numbers of males and females.

### Apparatus

Behavioral procedures were conducted in two identical two-compartment chambers, consisting of a white compartment and a black compartment separated by a guillotine door (PanLab, Harvard Apparatus). Shuttleboxes were made of Plexiglas and were 52 (L) × 26.5 (W) × 24 (H) cm. Each compartment was 26 (L) × 26.5 (W) × 24 (H) cm. The floor in each compartment consisted of a shock grid with 19 rods (0.3 cm diameter) spaced 1 cm apart. The Plexiglas box was divided into two compartments by an 8.0 (H) × 8.0 (W) cm guillotine door.

Each two-compartment chamber was housed in a separate room of the laboratory, creating two unique contexts. One room (i.e., the dark room) was lit by a red LED light and had a continuous 70 dB white noise. In this room, the chamber was cleaned with Windex. The other room (i.e., the light room) was lit by fluorescent lighting, had no additional ambient noise, and the chamber was cleaned with a lemon-scented all-purpose cleaner. Animal cages were covered during transport from the colony to the behavior rooms.

### Behavioral procedures

All animals were handled for 2 days prior to any behavioral procedures to acclimate to the experimenter. Behavioral procedures occurred during the light phase of the cycle and were separated by 24 h.

#### Training

Half of the animals received training in the dark room and the other half were trained in the light room. After they were transported to their designated room, they were held in the experimenter’s hand for 15 s, and then placed in the white side of the two-compartment chamber. The guillotine door was raised 30 s after placement in the chamber, allowing for movement to the other side of the chamber. Once the animal completely crossed from the white side of the chamber to the black side of the chamber, the guillotine door closed. Five seconds after closing, a 2-s footshock was delivered (either 0.7 mA or 1.5 mA depending on experiment). Thirty seconds following the footshock, the animal was removed from the chamber and returned to the main colony. The latency to cross from the white side to the black side of the chamber was recorded.

#### Testing

Testing occurred 24 h following training in the final experiment. Animals were tested in the same context as training or a shifted context and were split into four groups: animals that were trained and tested in the light context (group Light/Light), animals that were trained in the light context and tested in the dark context (group Light/Dark), animals that were trained and tested in the dark context (group Dark/Dark), and animals that were trained in the dark context and tested in the light context (group Dark/Light). Animals were taken to the appropriate testing context and were held in the experimenter’s hand for 15 s and then placed in the white side of the chamber. The guillotine door was raised after 30 s and remained open for 540 s. No shocks were delivered during testing. Latency to cross was measured and animals that did not cross were given a score of 540 s. After crossing or after 540 s (whichever came first), animals were removed from the chamber and returned to the colony.

### Tissue collection

Animals were anesthetized with an overdose of isoflurane and sacrificed 65 min following the beginning of their training session or their testing session depending on the experiment. Another group of animals were trained but did not undergo testing and remained in their homecage (group No Test) and were sacrificed with the groups that underwent testing. Two initial experiments examined how training, rather than testing, influenced expression of zif268 and PNN quantity by comparing an additional group that had no behavioral experience and remained in their homecage (group No Train) to a group that was sacrificed after training. All brains were removed and immediately flash frozen for subsequent tissue analysis.

### Immunofluorescence

Tissue from the experiments was sliced in 40-micron sections and mounted onto charged slides for immunofluorescence. Coordinates for the BLA were AP: −2.6, ML: ±5.0, DV: −7.0 and 4 bilateral images were taken per rat. Coordinates for the anterior RSC were AP: −2.6, ML: ±0.5, DV: −1.5. Coordinates for the posterior RSC were AP: −5.6, ML: ±1.0, DV: −1.5. Six bilateral images were taken for each retrosplenial subregion. For all IF experiments, exceptions were made in cases where tissue was damaged.

#### Zif268

**Figure 1:**
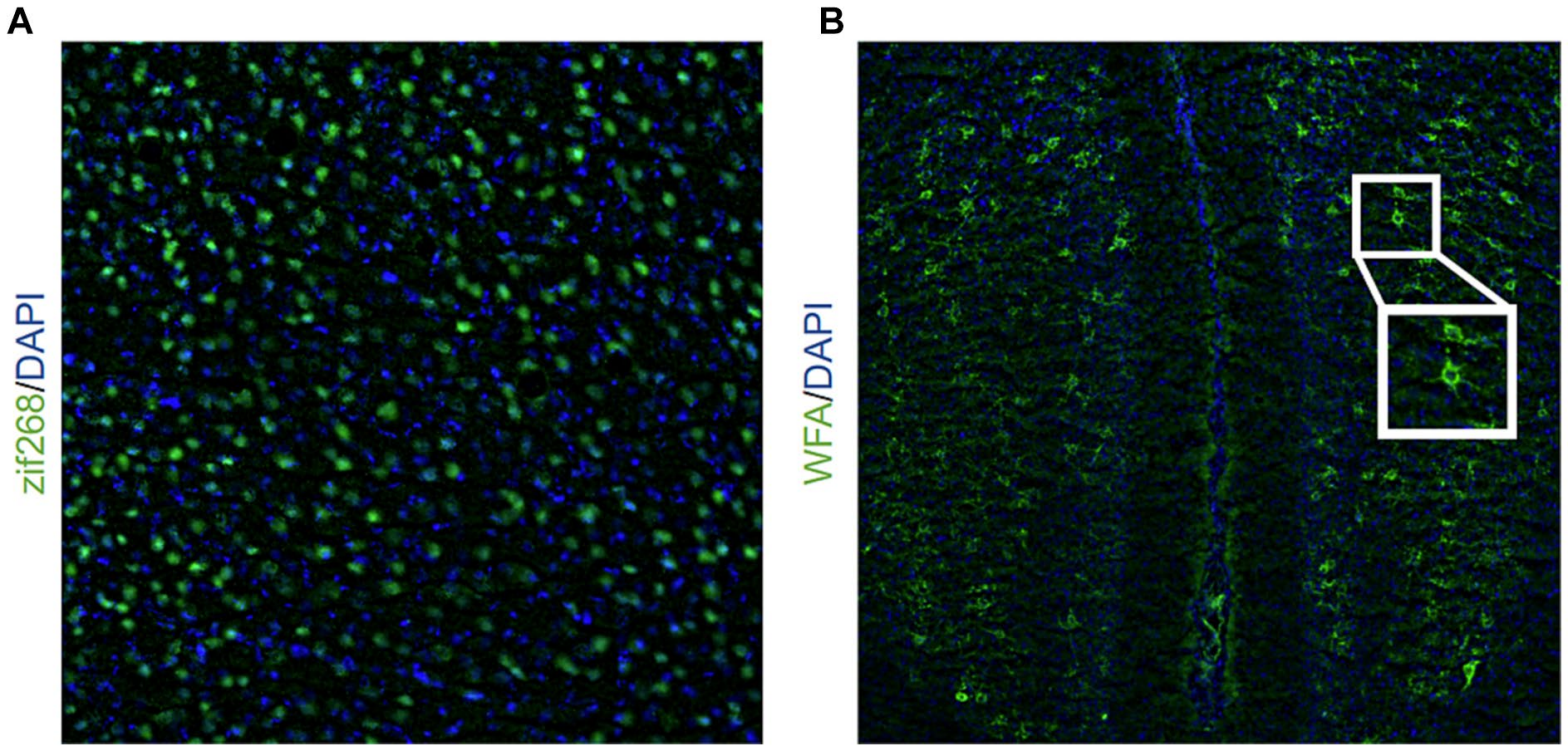
Representative image of **(A)** zif268 staining (20X objective lens) in green and DAPI in blue and **(B)** WFA staining (10X objective lens) in green and DAPI in blue. The inlet shows a representative example of two PNNs indicated by WFA staining.

Slides were fixed in 10% buffered formalin before being rehydrated in wash buffer (PBS + 0.05% Tween-20) and permeabilized (PBS + 0.3% Triton X) for 15 min and incubated in blocking solution (PBS + 0.7% NGS) for 30 min. Slides incubated in zif268/EGR-1 antibody (Cell Signaling, 1:400) solution (PBS + 0.3% Triton X + 5% NGS) overnight at 4°C. The next day, tubes incubated at room temperature for 2 h before incubation in secondary antibody solution for 2 h. Slides were rinsed with wash buffer, a DAPI counterstain was applied, and then they were coverslipped. Images (bilateral) were captured from the BLA, aRSC, and pRSC on the Leica THUNDER imager system using a 20X objective acquired using LAS-X software (Leica). Images were exported as 12-bit TIFF files and converted to a binary image via Gaussian filtering (sigmas: 3, 6) then quantified using the “Analyze Particles” plugin in ImageJ. Zif268 activity was normalized as a proportion of DAPI present in the same section. Zif268 expression in all experimental groups was normalized to a control condition who received all behavioral procedures up until the day of sacrifice, when they remained in their homecage. This way, we are able to determine changes in event-related activity relative to a group who received no experimental event (either memory formation or retrieval) on the day in which activity was examined. An example image of zif268 staining is depicted in Figure 1A. Example images from each experimental group are shown in Supplementary Figures S1, S3, S5.

#### WFA staining for PNNs

Slides were washed with PBS three times for 5 min, permeabilized (PBS+ 0.5% Triton X-100) for 30 min, and again washed with PBS for 10 min. They incubated in carbo-free blocking solution (0.1% Triton + CFBS) for 60 min before they were stained with the biotinylated *Wisteria floribunda* Agglutinin (WFA) primary (Sigma Aldrich, 1:2,000), a marker of PNNs (Slaker et al., 2016), and incubated overnight at 4° C. The next day, tubes were removed from the fridge and remained at room temperature for 2 h before they were washed with PBS 3 times for 10 min. The slides incubated in the streptavidin-conjugated Dylight 488 donkey anti-rabbit secondary (1:200 in PBS) for 2 h. Slides were rinsed with PBS 4 times for 10 min, a DAPI counterstain was applied, and then they were coverslipped. Images were captured from the BLA, aRSC, and pRSC on the Leica THUNDER imager system using a 10X objective (Leica). For each slice where the brain region was present, we captured two BLA images (one for the right hemisphere and one for the left hemisphere) and one RSC image over the midline (see Figure 1B). WFA images were exported as 12-bit TIFF images. PNNs, indicated by WFA staining, were hand-counted by experimenters blinded to conditions. An example image of PNNs is depicted in Figure 1B. Example images from each experimental group are shown in Supplementary Figures S2, S4, S6.

### Data analysis

All data were analyzed using SPSS (Version 29) with ANOVAs or *t*-tests as appropriate. Alpha was set to 0.05 and planned comparisons were used to follow-up on main effects or interactions to compare experimental groups to controls. One animal was excluded due to receiving multiple shocks during training. Final group sizes for the experiment that used a weak shock during training were: Homecage controls, *n* = 8; Training, *n* = 8. In the experiment where animals were trained using a strong shock, final group sizes were: Homecage controls, *n* = 6; Training, *n* = 6. Final group sizes for animals tested for retrieval of an inhibitory avoidance memory were: Training Only (Control), *n* = 12; Light/Light, *n* = 9; Light/Dark, *n* = 8; Dark/Dark, *n* = 7; Dark/Light, *n* = 8. Individual data points on each graph represent individual animals. Figures were created using Graphpad Prism (Version 10).

## Results

### Training with a weak shock (0.7 mA) increases zif268 activity in the BLA, aRSC, and pRSC but does not reduce PNN quantity

**Figure 2:**
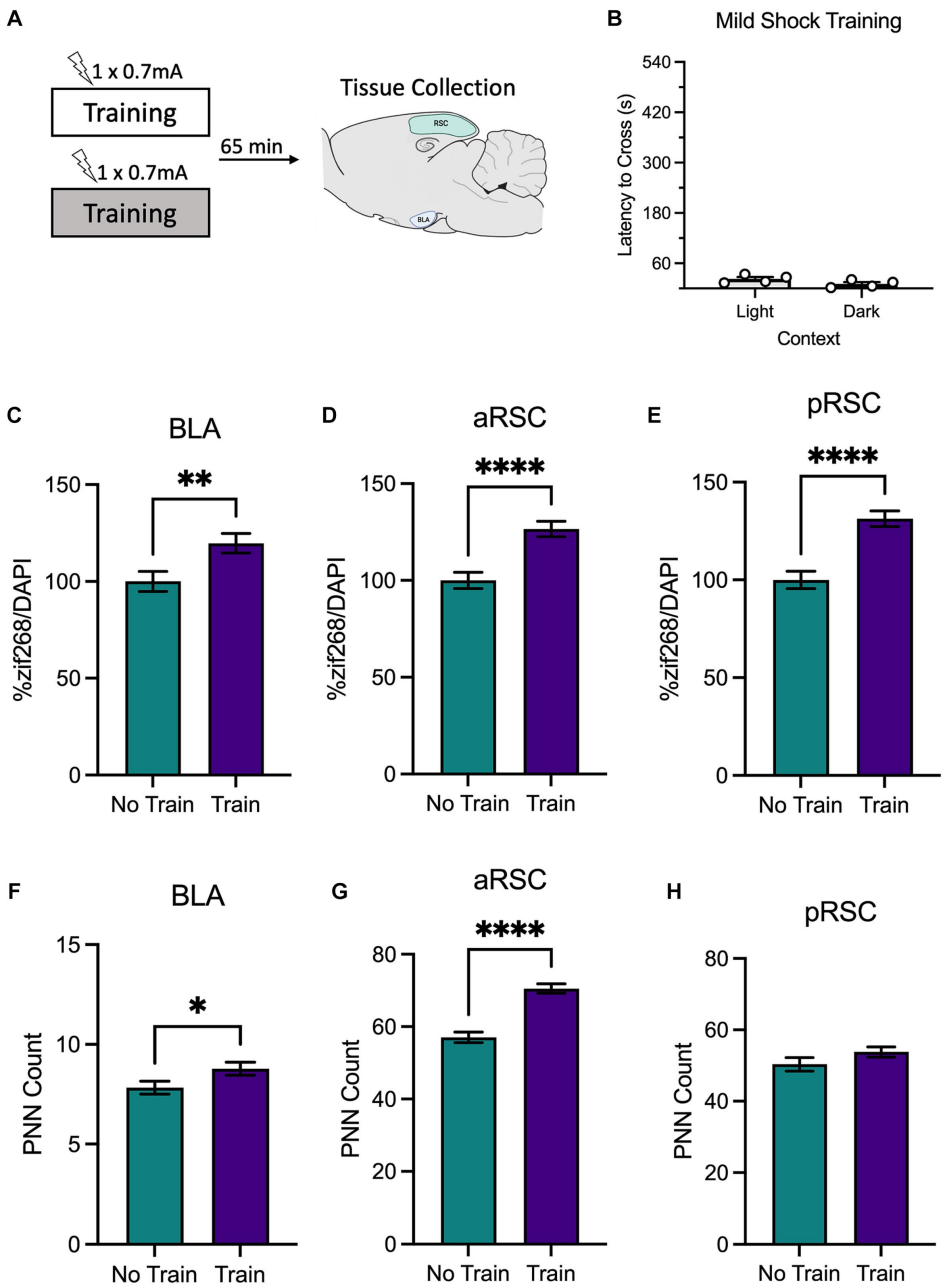
**(A)** Schematic depicting behavioral design and tissue collection. Animals received inhibitory avoidance training with a mild shock (0.7 mA) in a light context or a dark context. Animals in experimental conditions were sacrificed 65 min following training with another group of animals that remained in their homecage and served as the molecular control (Group No Train). **(B)** Behavioral results from animals that received training in the light context and the dark context. Animals that received training (Group Train), regardless of context, showed an increase in zif268 activity in the **(C)** BLA, **(D)** aRSC, and **(E)** pRSC compared to their No Train counterparts. Training did increase PNNs in the **(F)** BLA and **(G)** aRSC, but not **(H)** pRSC. *p < 0.05, **p < 0.01, ***p < 0.001, ****p < 0.001.

Here, we aimed to determine if training with a weak shock would produce changes in neural activity (experimental design is depicted in Figure 2A). Unpublished work from our lab has shown that a weak shock during training is not enough to produce an avoidance response during testing, suggesting that a 0.7 mA shock is not enough for animals to form a robust memory. Because of this, we predicted that a weak shock would not cause a reduction in PNNs.

A between-subjects *t*-test found that animals trained with a mild shock (Figure 2B) did not differ in latency to cross when training occurred in the light context or the dark context, *t*_(6)_ = 1.84, *p* = 0.115, as expected. Animals were sacrificed immediately after training.

We then examined zif268 activity as a proxy for neural activity in the BLA, aRSC, and pRSC. Animals trained with a weak shock had elevated levels of zif268 in the BLA (Figure 2C), *t*_(121)_ = 2.73, *p* = 0.007, aRSC (Figure 2D), *t*_(190)_ = 4.57, *p* < 0.0001, and pRSC (Figure 2E), *t*_(179)_ = 5.18, *p* < 0.0001. An increase was also observed in PNN amount in the aRSC (Figure 2G), *t*_(95)_ = 6.90, *p* < 0.0001, and in the BLA (Figure 2F), *t*_(146)_ = 2.08, *p* = 0.039, but not the pRSC (Figure 2H), *t*_(77)_ = 1.48, *p* = 0.142. Together this suggests that training with a weak shock is enough to increase activity in the BLA, aRSC, and pRSC, but does not induce decreases in PNN amount characteristic of memory formation.

### Training with a strong shock (1.5 mA) increases neural activity in the BLA, aRSC, and pRSC and reduces PNNs in the aRSC

**Figure 3:**
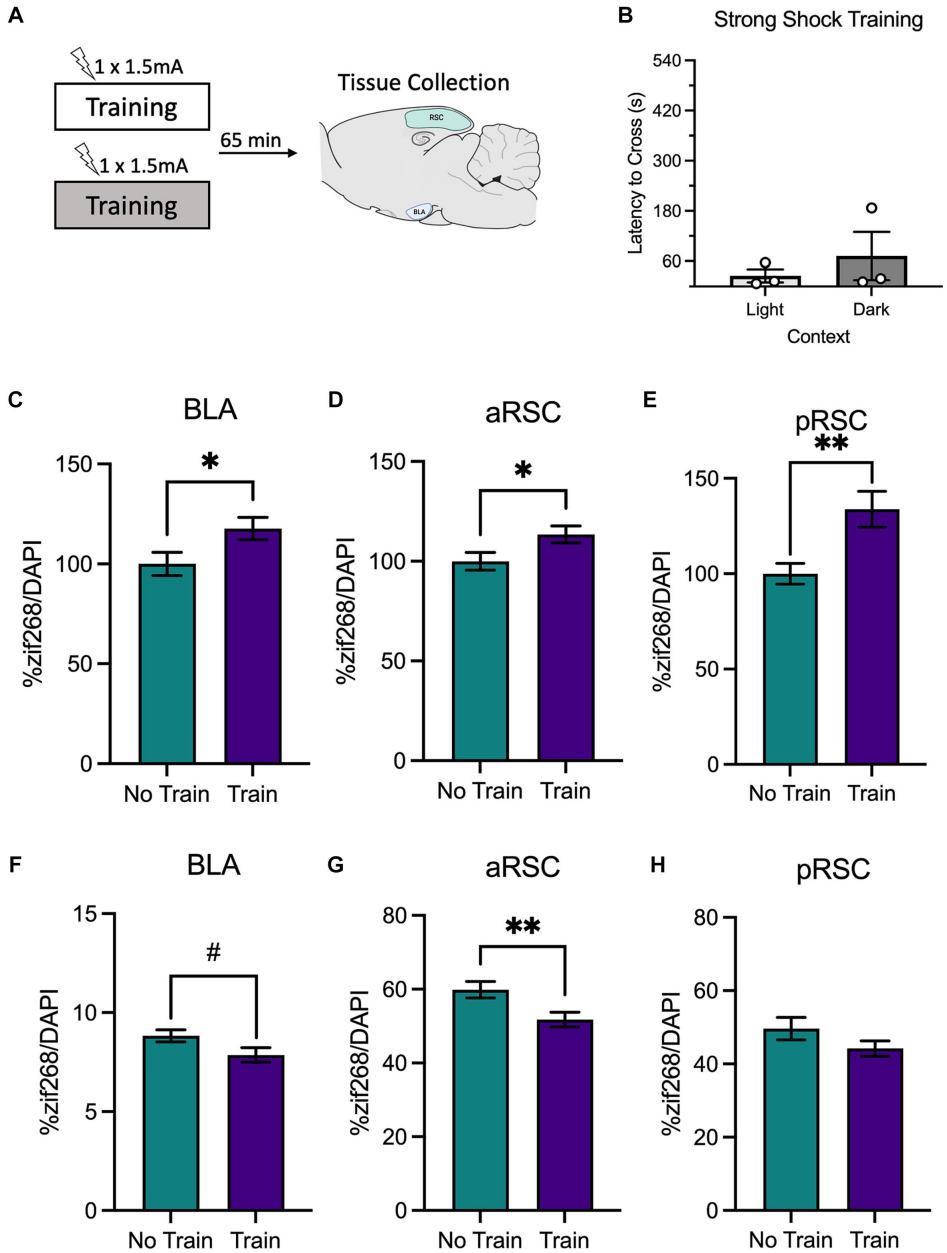
**(A)** Schematic depicting behavioral design and tissue collection. Animals received inhibitory avoidance training with a strong shock (1.5 mA) in a light context or a dark context. Animals in the experimental groups were sacrificed 65 min following their training session along with a group of animals that did not receive training (Group No Train) and served as the molecular control. **(B)** Latencies to cross did not differ between animals trained in the light context or the dark context. There was elevated zif268 activity in the **(C)** BLA, **(D)** aRSC and **(E)** pRSC of animals in the animals that received training (Group Train) compared to the homecage controls (Group No Train). While a numerical decrease in PNNs was observed in the **(F)** BLA, it did not reach significance. In the **(G)** aRSC, there was a decrease in PNN count in animals that received training. **(H)** Differences were not observed in PNN quantity in the pRSC between groups. ^#^*p* < 0.10, *p < 0.05, **p < 0.01.

Next, we used a similar design to determine changes in neural activity following training with a strong shock, which in our hands produces a strong avoidance response during testing (experimental design is depicted in Figure 3A). Again, animals did not differ in their latencies to cross during training based on context (light or dark), *t*_(4)_ = 0.79, *p* = 0.473 (Figure 3B). As before, animals were sacrificed immediately after training, and we examined zif268 and PNNs.

Similar to the findings from the weak shock experiment, we observed elevated zif268 expression in the BLA (Figure 3C), *t*_(105)_ = 2.19, *p* = 0.031, aRSC (Figure 3D), *t*_(135)_ = 2.17, *p* = 0.032, and pRSC (Figure 3E), *t*_(124)_ = 3.19, *p* = 0.002. While there appeared to be a decrease in quantity of PNNs in the BLA (Figure 3F) this failed to reach statistical significance, *t*_(122)_ = 1.96, *p* = 0.053. In the aRSC (Figure 3G) PNNs were reduced in the train group, *t*_(58)_ = 2.70, *p* = 0.009. These differences were not observed in the pRSC (Figure 3H), *t*_(43)_ = 1.51, *p* = 0.139. This indicates that training with a strong shock increases neural activity and reduces PNNs in the BLA and aRSC, which may be a contributing factor to synaptic plasticity associated with memory formation.

### Animals tested in the light maintained context-specificity and animals tested in the dark generalized their avoidance behavior

**Figure 4:**
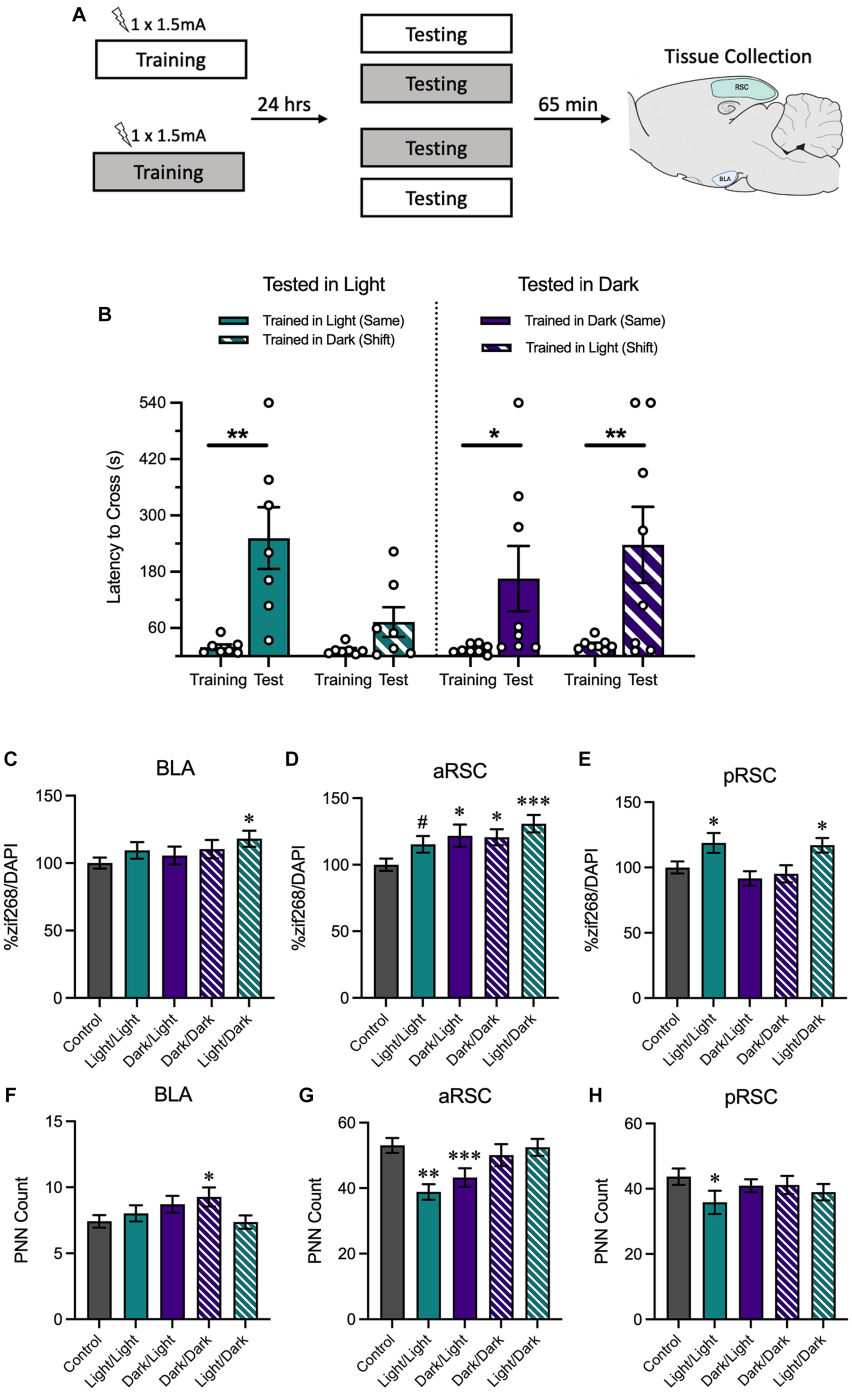
**(A)** Schematic depicting behavioral design and tissue collection. Animals received inhibitory avoidance training with a strong shock (1.5 mA) in either the light context or the dark context. Animals were tested 24 h later in the same context they received training in (Light/Light, Dark/Dark) or in a shifted context (Light/Dark, Dark/Light). Sixty-five minutes following testing sessions animals were sacrificed with another group of animals that received training but not testing (Group No Test). **(B)** Animals tested in the light context displayed longer latencies to cross when tested in the same context (Light/Light) and shorter latencies to cross when tested in a novel context (Dark/Light). Meanwhile, animals tested in the dark context exhibited similarly-long latencies to cross in both the same (Dark/Dark) and the novel context (Light/Dark). **(C)** In the BLA, animals trained in the light and tested in the dark showed an increase in zif268. **(D)** In the aRSC, there was an increase in zif268 activity in all tested groups, but this was only a numerical increase in the animals trained and tested in the light. **(E)** Only animals trained in the light showed increases in zif268 activity in the pRSC. **(F)** While only group Dark/Dark showed increases in the BLA and **(H)** only group Light/Light showed decreased PNN quantity in the pRSC, **(G)** there was a decrease in PNNs in the aRSC of animals tested in the light. ^#^*p* < 0.10, *p < 0.05, **p < 0.01, ***p < 0.001.

A final experiment assessed how memory retrieval in either the same context as training or a different context from training influenced behavioral performance as well as neural activity in the retrosplenial cortex and basolateral amygdala (experimental design in Figure 4A).

We assessed latency to cross from the white compartment to the black compartment during training and testing with a 2 (Time Point: Training, Testing) × 2 (Training Context: Light, Dark) × 2 (Testing Context: Light, Dark) ANOVA (Figure 4B). We found that there was a main effect of time, *F*_(1, 26)_ = 23.54, MSE = 17030.37, *p* < 0.001*, h_p_*^2^ = 0.48, and a trend of training context, *F*_(1, 26)_ = 4.05, MSE = 16391.78, *p* = 0.055, *h_p_*^2^ = 0.49. No other interactions or main effects were significant, largest *F* = 3.03, *p* = 0.094. Planned comparisons revealed that animals that were both trained and tested in the light context increased their latency between training and testing, *p* = 0.003, indicating that animals showed strong avoidance responding. Conversely, there was no increase between training and testing in animals trained in the dark context and tested in the light context, *p* = 0.401, suggesting that animals in this condition successfully discriminated between the two contexts. Similar to what was observed when animals received both training and testing in the light context, animals trained and tested in the dark showed an increase between training and testing, *p* = 0.029. However, this was also true of animals that were trained in the light and tested in the dark, *p* = 0.003, suggesting that despite being tested in a novel context, they showed a robust avoidance response. Altogether, these data indicate that animals tested in the light were able to maintain context-specificity and only avoid the shock-associated side of the chamber when they were placed in the same context as training; animals that were tested in the dark were more likely to avoid the shock-associated side of the chamber, even if they were tested in a novel context.

Next, we measured neural activity using zif268 in BLA, aRSC, and pRSC. A one-way ANOVA found between-group differences in the aRSC (Figure 4D), *F*_(4,493)_ = 4.05, *p =* 0.003. Follow-up comparisons revealed an increase in zif268 expression in the aRSC of all tested groups compared to the untested controls (Light/Dark, *p* < 0.001; Dark/Dark, *p* = 0.013; Dark/Light, *p* = 0.011), except for group Light/Light which did not show a difference, *p* = 0.067. A between-groups difference was revealed in the pRSC (Figure 4E), *F*_(4,430)_ = 4.16, *p =* 0.003. Here, this difference was primarily driven by an increase in activity in groups Light/Light (*p* = 0.019) and Light/Dark (*p* = 0.034) relative to controls. Both groups trained in the dark no increase in activity in the pRSC, smallest *p* = 0.306. While no overall group differences were observed in the BLA (Figure 4C), *F*_(4,398)_ = 1.68, *p =* 0.154, the Light/Dark group did show an increase (*p* = 0.012); the other groups did not (smallest *p* = 0.196).

Using the same ANOVA, we also found differences in PNN counts in the aRSC (Figure 4G), *F*_(4,217)_ = 5.42, *p <* 0.001. Follow-up comparisons revealed that animals tested in the light (groups Light/Light, *p <* 0.001, and Dark/Light, *p =* 0.008), had a reduction in PNNs compared to the no test controls. Differences were not observed in the pRSC (Figure 4H), *F*_(4,177)_ = 1.87, *p =* 0.318, (although group Light/Light showed a decrease relative to controls, *p* = 0.039), or in the BLA (Figure 4F), *F*_(4,319)_ = 1.92, *p =* 0.108 (although group Dark/Dark showed an increase relative to controls, *p* = 0.025).

### Behavioral performance, and not *a priori* group assignment *per se*, corresponds with changes in neural activity

**Figure 5:**
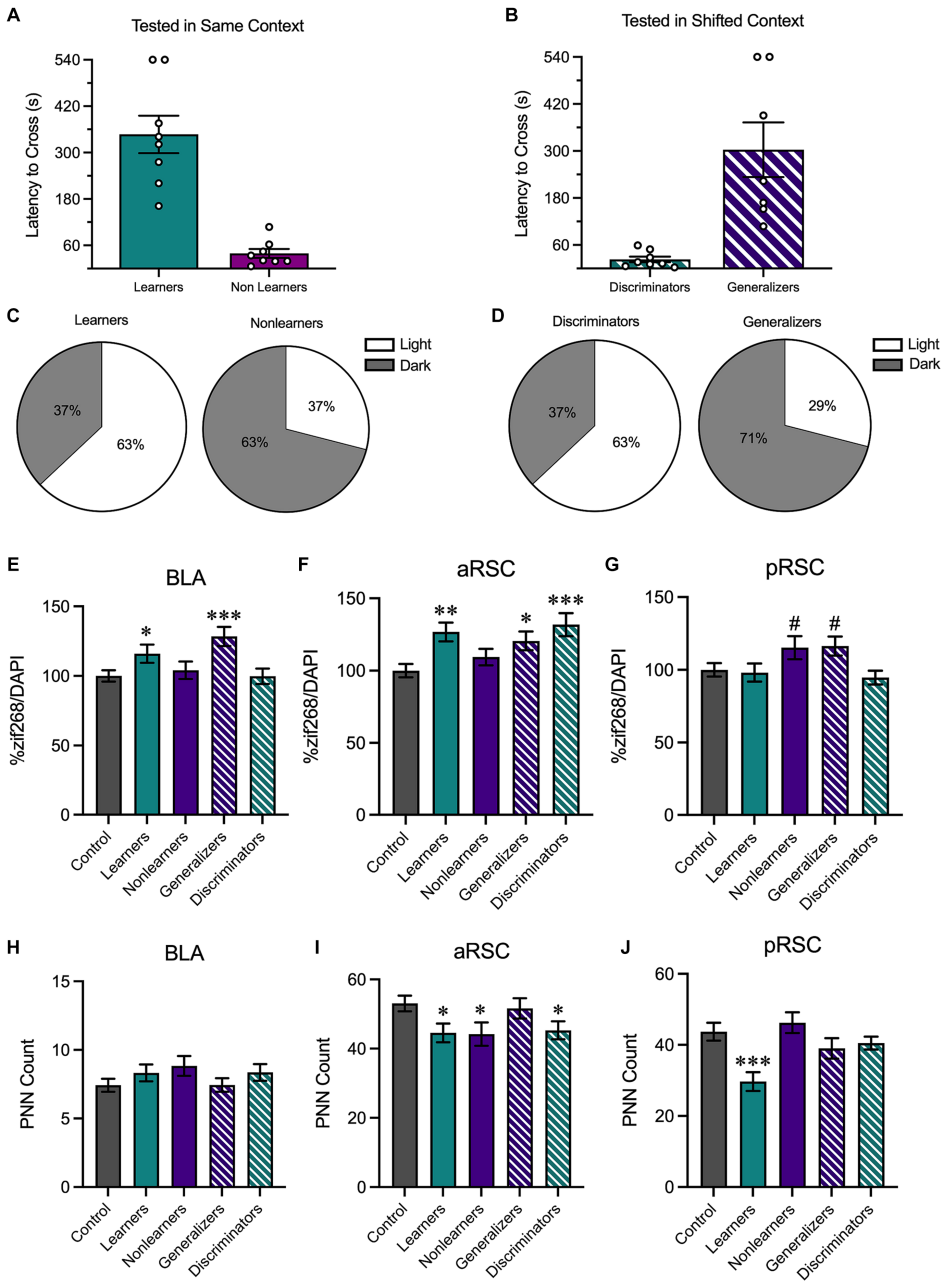
We then performed a median split analysis to determine how behavioral performance influenced neural activity. **(A)** Of animals tested in the same context, animals with higher latencies to cross were classified as learners and those with shorter latencies to cross were classified as nonlearners. **(B)** Of animals tested in a shifted context, those with shorter latencies to cross were classified as discriminators and those with longer latencies to cross were classified as generalizers. **(C)** 63% of learners were tested in the light context and 37% were tested in the dark context; 63% of nonlearners were tested in the dark context and 37% were tested in the light context. **(D)** 63% of discriminators were tested in the light context and 37% were tested in the dark context; 71% of generalizers were tested in the dark context and 29% were tested in the light context. **(E)** In the BLA, there was an increase in zif268 in animals that showed increased avoidance behaviors (i.e., learners and generalizers). **(F)** In the aRSC, there was an increase in neural activity in all groups except for nonlearners. **(G)** In the pRSC, while there were numerical increases in nonlearners and generalizers, no group differences were observed. **(H)** While no differences were observed in PNN counts in the BLA, **(I)** there was a decrease in PNN quantity in the learners, discriminators, and nonlearners in the aRSC and **(J)** a reduction in PNNs in the pRSC of learners. *p < 0.05, **p < 0.01, ***p < 0.001.

We then performed a median split analysis on the behavioral data obtained during the testing phase. All animals tested in the same environment (regardless of context identity) were rank ordered based on latency to cross to the dark side of the chamber during the testing phase. Animals who had higher latencies to cross were labeled as learners reflecting that they performed the contextually-appropriate avoidance response; animals in the bottom half were labeled as nonlearners (Figure 5A). A similar analysis was performed on animals tested in a shifted environment. Here, animals with shorter latencies were classified as discriminators and those with longer latencies were classified as generalizers (Figure 5B). Although they displayed opposite behavioral patterns, both learners and discriminators shared a vital commonality: they exhibited contextually-appropriate responses. Similarly, learners and generalizers have a shared characteristic in that both show a robust avoidance response. While 63% of the learners and 63% of the discriminators were tested in the light context (Figure 5C), 63% of the nonlearners and 71% of the generalizers were tested in the dark context (Figure 5D). This aligns with the results reported in Figure 4 and suggests that contextually-appropriate responding was more likely in animals tested in the light context.

We then used these new behavioral categories to assess zif268 activity in the BLA, aRSC, and pRSC. In the BLA (Figure 5E), a one-way ANOVA found group differences, *F*_(4,398)_ = 4.54, *p* = 0.001. Learners (*p* = 0.040) and generalizers (*p <* 0.001) had increased zif268 expression relative to controls, suggesting that an increase in zif268 activity corresponds with overall avoidance behavior. This is supported by the fact that similar increases relative to controls were not found in nonlearners and discriminators, smallest *p* = 0.587, who do not show a strong avoidance response. A similar pattern was observed in the aRSC (Figure 5F), *F*_(4,493)_ = 5.039, *p* < 0.001. Learners (*p* = 0.001), discriminators (*p* < 0.001), and generalizers (*p* = 0.016) all showed increases relative to controls. This was not the case in nonlearners, *p* = 0.257. While the ANOVA was significant in the pRSC (Figure 5G), *F*_(4,430)_ = 2.67, *p* = 0.032, no groups differed from controls (Learners: *p* = 0.811, Nonlearners: *p* = 0.056, Generalizers: *p* = 0.051, Discriminators: *p* = 0.506).

We then used the same one-way ANOVA to evaluate PNN counts in the BLA (Figure 5H), aRSC (Figure 5I), and pRSC (Figure 5J). In the aRSC there was an effect of group, *F*_(4,217)_ = 2.57, *p* = 0.039, and our *a priori* planned comparisons revealed a significant decrease in PNNs in learners (*p* = 0.023) discriminators (*p* = 0.030) and nonlearners (*p* = 0.019) compared to no test controls. This was not the case in generalizers (*p* = 0.713). We identified group differences in the pRSC, *F*_(4,177)_ = 5.24, *p* < 0.001. This difference was primarily driven by a reduction of PNNs in learners, *p* < 0.001, but no other groups, smallest *p* = 0.196. We did not observe any changes in quantity in the BLA, *F*_(4,319)_ = 1.16, *p* = 0.328.

## Discussion

Here we used an inhibitory avoidance paradigm to investigate how generalization of an aversive memory to a novel context corresponded with changes in zif268 expression and perineuronal net quantity within the retrosplenial cortex, a region that has been widely-hypothesized to play a role in systems-level consolidation that contributes to memory generalization. We found that while training with a 0.7 mA shock (which in our hands is insufficient to produce a robust avoidance memory) increases neural activity in the BLA and both subregions of the RSC, it does not reduce PNN quantity. Heightened cellular activity marked by increased zif268 expression has been linked to active memory processes (Hoffman et al., 2015; Trask et al., 2021a,b). However, the absence of any concurrent changes in PNN count following the weak shock suggests that increased cellular activity is not necessarily sufficient to produce changes in synaptic plasticity associated with memory formation (Carulli et al., 2020). In line with this interpretation, we observed that training with a strong shock that typically produces a strong avoidance memory corresponded with increases in zif268 activity and a concurrent decrease in PNNs in the aRSC. However, it should be noted that any behavioral experience, including placement in a chamber, can be enough to drive increases in zif268 expression in the RSC (Asok et al., 2013) so the present results may therefore be indicative of any behavioral experience rather than the inhibitory avoidance memory acquisition, *per se*.

Next, we found that animals tested in a light context maintained a context-specific avoidance response, showing more avoidance when they were tested in the same context as training than when they were tested in a shifted context, aligning with earlier studies which demonstrated context-specificity of inhibitory avoidance when tested the day after training (e.g., MacArdy and Riccio, 1995; Zhou and Riccio, 1996; McAllister and McAllister, 2006). However, when animals were tested in the dark context, avoidance was high regardless of where the animals received training, demonstrating an asymmetrical generalization between these environments, similar to the results reported in Oleksiak et al. (2021). This observation was further supported by a median split analysis on the behavioral data obtained during the testing phase of all animals tested in either the same context or a shifted context, where the majority of animals tested in the light context were learners and discriminators, whereas those tested in the dark context were nonlearners and generalizers.

One possibility for this finding is that this work was conducted during the light cycle. The increase in avoidance behavior in the dark may be attributed to a shift in the animals’ natural expectations influenced by their circadian rhythm, potentially causing a stress response. Future work aiming to determine if this is the case should include animals tested in the light during the dark phase of the cycle. Another possible explanation is that inhibitory avoidance depends on the species-specific tendency to prefer darkness and therefore cross from the white side of the chamber to the black side more readily; here, testing in the dark may have reduced this tendency as the room was already dark. This second explanation, however, is less likely as animals tested in a two-way signaled active avoidance paradigm showed a similar impact of testing in the darkness on avoidance responding (Oleksiak et al., 2021).

The retrosplenial cortex has a well-known role in contextual memory formation and retrieval (Kwapis et al., 2015; Todd and Bucci, 2015; Todd et al., 2016, 2019; Keene and Bucci, 2021; Trask et al., 2021a,b; Trask and Helmstetter, 2022), with the anterior and posterior subregions having distinct but complementary roles. While the aRSC is needed for the acquisition of information related to a CS, or an event, the pRSC is needed for the acquisition of context-related information (Trask et al., 2021a). Activity in the RSC may also facilitate systems-level consolidation. While activity within the hippocampus and BLA is necessary for memory formation and recent memory retrieval (Maren and Fanselow, 1995, 1996; McIntyre et al., 2005; Roesler et al., 2021), the RSC may mediate the transfer from recent to remote memory, where retrieval is less dependent on the hippocampus and more dependent on the anterior cingulate cortex (de Sousa et al., 2019; Trask et al., 2021c). Interestingly, the aRSC seems to share more dense reciprocal connections with the anterior cingulate cortex and the pRSC is more highly connected with the DH (Sugar et al., 2011), which is necessary for contextual components of learning. This DH-pRSC connectivity may partially explain changes in the pRSC only when trained in the light. Consistent with our hypothesis and other findings implicating the aRSC in memory, we found an increase in neural activity in the aRSC of animals characterized as learners, generalizers, and discriminators – all groups except for the nonlearners. Overall levels of avoidance behaviors, as those seen in learners and generalizers, corresponded with increased activity in the BLA, similar to other work demonstrating a correlation between BLA activity and heightened fear states (Hoffman et al., 2015; Bonanno et al., 2023). While it was just a trend, increased zif268 in the pRSC during generalization is consistent with previous research demonstrating increased retrosplenial activity in new or novel environments (Asok et al., 2013). Similarly, an increase in zif268 expression in the nonlearners may correspond with a lack of memory for that environment and encoding of unremembered contextual information.

Changes in memory consolidation have also been associated with modifications in perineuronal nets. Typically, it is believed that PNNs stabilize following memory consolidation (Wang and Fawcett, 2012; Sorg et al., 2016). Here, we hypothesized that contextual discrimination would align with stabilization of PNNs in the pRSC given its role in context-specific information, but that generalization of an inhibitory avoidance memory would correspond with a greater number of PNNs in the aRSC and BLA. While we found no changes in the BLA, we found a reduction of PNNs in the aRSC corresponded with memory retrieval seen in animals classified as learners, nonlearners, and discriminators. This reduction was also seen in the pRSC, but only in learners. One possible explanation for the lack of changes in PNN quantity in both the aRSC and pRSC in generalizers might suggest that, in line with our original hypothesis, generalized memory corresponds with PNN stabilization characteristic of a consolidated memory. The reductions observed in PNN quantities in other groups may be indicative of synaptic plasticity associated with reconsolidation-like processes where a memory becomes labile following retrieval (Nader et al., 2000; Einarsson and Nader, 2012; for review, see Sara, 2000), suggesting that recalling a specific one-day-old memory induces synaptic plasticity associated with memory retrieval (e.g., Nader et al., 2000; Parsons et al., 2006; Lee et al., 2008; Hong et al., 2013; Jarome et al., 2015; Kwapis et al., 2017). In the nonlearners, this might correspond with new learning, but this possibility has yet to be explored.

Further, it should be noted that the present experiments did not delineate between activity in the granular and dysgranular layers of the RSC (e.g., Sugar et al., 2011), and instead focused on the rostrocaudal subdivisions based on their hypothesized role in systems consolidation through different anatomical connections (e.g., Trask et al., 2021c). However, given the observed dissociable roles for the granular and dysgranular layers of the retrosplenial cortex in using available light cues (e.g., Aggleton et al., 2021) follow-up experiments should examine how the anterior and posterior granular and dysgranular layers contribute to memory generalization that occurs when testing without light cues.

In conclusion, we have identified differences in neural activity within the retrosplenial cortex that correspond with memory specificity or generalization. Future work will examine how PNNs change following a long delay between training and retrieval, when memory has likely undergone systems consolidation as well as how manipulating neural function within these regions impacts memory acquisition and generalization.

## Data availability statement

The raw data supporting the conclusions of this article will be made available by the authors, without undue reservation.

## Ethics statement

The animal study was approved by Purdue University Institutional Animal Care and Use Committee. The study was conducted in accordance with the local legislation and institutional requirements.

## Author contributions

EM: Conceptualization, Data curation, Formal analysis, Writing – original draft, Writing – review & editing, Investigation, Methodology. PR: Investigation, Writing – review & editing. KG: Investigation, Writing – review & editing. ST: Conceptualization, Data curation, Formal analysis, Funding acquisition, Resources, Supervision, Writing – original draft, Writing – review & editing.

## References

[ref1] Aggleton J. P. Yanakieva S. Sengpiel F. Nelson A. J. (2021). The separate and combined properties of the granular (area 29) and dysgranular (area 30) retrosplenial cortex. Neurobiol. Learn. Mem. 185:107516. doi: 10.1016/j.nlm.2021.107516, PMID: 34481970

[ref2] Anagnostaras S. G. Maren S. Fanselow M. S. (1999). Temporally graded retrograde amnesia of contextual fear after hippocampal damage in rats: within-subjects examination. J. Neurosci. 19, 1106–1114. doi: 10.1523/JNEUROSCI.19-03-01106.1999, PMID: 9920672 PMC6782148

[ref3] Asok A. Schreiber W. B. Jablonski S. A. Rosen J. B. Stanton M. E. (2013). Egr-1 increases in the prefrontal cortex following training in the context preexposure facilitation effect (CPFE) paradigm. Neurobiol. Learn. Mem. 106, 145–153. doi: 10.1016/j.nlm.2013.08.006, PMID: 23973447 PMC3852177

[ref4] Bonanno G. R. Met Hoxha E. Robinson P. K. Ferrara N. C. Trask S. (2023). Fear reduced through unconditional stimulus deflation is behaviorally distinct from extinction and differentially engages the amygdala. Biol. Psychiatry Glo. Open Sci. 3, 756–765. doi: 10.1016/j.bpsgos.2023.01.001, PMID: 37881558 PMC10593882

[ref5] Bozon B. Kelly A. Josselyn S. A. Silva A. J. Davis S. Laroche S. (2003). MAPK, CREB and zif268 are all required for the consolidation of recognition memory. Philos. Trans. R. Soc. Lond. B Biol. Sci. 358, 805–814. doi: 10.1098/rstb.2002.1224, PMID: 12740127 PMC1693143

[ref6] Carulli D. Broersen R. de Winter F. Muir E. M. Mešković M. de Waal M.. (2020). Cerebellar plasticity and associative memories are controlled by perineuronal nets. Proc. Natl. Acad. Sci. 117, 6855–6865. doi: 10.1073/pnas.1916163117, PMID: 32152108 PMC7104182

[ref7] Corcoran K. A. Donnan M. D. Tronson N. C. Guzmán Y. F. Gao C. Jovasevic V.. (2011). NMDA receptors in retrosplenial cortex are necessary for retrieval of recent and remote context fear memory. J. Neurosci. 31, 11655–11659. doi: 10.1523/JNEUROSCI.2107-11.2011, PMID: 21832195 PMC3159389

[ref8] de Sousa A. F. Cowansage K. K. Zutshi I. Cardozo L. M. Yoo E. J. Leutgeb S.. (2019). Optogenetic reactivation of memory ensembles in the retrosplenial cortex induces systems consolidation. Proc. Natl. Acad. Sci. 116, 8576–8581. doi: 10.1073/pnas.1818432116, PMID: 30877252 PMC6486739

[ref9] Einarsson E. Ö. Nader K. (2012). Involvement of the anterior cingulate cortex in formation, consolidation, and reconsolidation of recent and remote contextual fear memory. Learn. Mem. 19, 449–452. doi: 10.1101/lm.027227.112, PMID: 22984282

[ref11] Frankland P. W. Bontempi B. (2005). The organization of recent and remote memories. Nat. Rev. Neurosci. 6, 119–130. doi: 10.1038/nrn1607 15685217

[ref12] Frankland P. W. Bontempi B. Talton L. E. Kaczmarek L. Silva A. J. (2004). The involvement of the anterior cingulate cortex in remote contextual fear memory. Science 304, 881–883. doi: 10.1126/science.1094804, PMID: 15131309

[ref13] Hoffman A. N. Parga A. Paode P. R. Watterson L. R. Nikulina E. M. Hammer R. P. Jr.. (2015). Chronic stress enhanced fear memories are associated with increased amygdala zif268 mRNA expression and are resistant to reconsolidation. Neurobiol. Learn. Mem. 120, 61–68. doi: 10.1016/j.nlm.2015.02.004, PMID: 25732249 PMC4397163

[ref14] Hong I. Kim J. Kim J. Lee S. Ko H. G. Nader K.. (2013). AMPA receptor exchange underlies transient memory destabilization on retrieval. Proc. Natl. Acad. Sci. 110, 8218–8223. doi: 10.1073/pnas.1305235110, PMID: 23630279 PMC3657785

[ref15] Jarome T. J. Ferrara N. C. Kwapis J. L. Helmstetter F. J. (2015). Contextual information drives the reconsolidation-dependent updating of retrieved fear memories. Neuropsychopharmacology 40, 3044–3052. doi: 10.1038/npp.2015.161 26062788 PMC4864640

[ref16] Jasnow A. M. Lynch J. F. III Gilman T. L. Riccio D. C. (2017). Perspectives on fear generalization and its implications for emotional disorders. J. Neurosci. Res. 95, 821–835. doi: 10.1002/jnr.23837 27448175

[ref17] Katche C. Dorman G. Gonzalez C. Kramar C. P. Slipczuk L. Rossato J. I.. (2013). On the role of retrosplenial cortex in long-lasting memory storage. Hippocampus 23, 295–302. doi: 10.1002/hipo.22092, PMID: 23355414

[ref18] Keene C. S. Bucci D. J. (2021). Contributions of the retrosplenial and posterior parietal cortices to cue-specific and contextual fear conditioning. Behav. Neurosci. 135, 693–701. doi: 10.1037/bne0000435 34871020

[ref19] Kessler R. C. Berglund P. Demler O. Jin R. Merikangas K. R. Walters E. E. (2005). Lifetime prevalence and age-of-onset distributions of DSM-IV disorders in the National Comorbidity Survey Replication. Arch. Gen. Psychiatry 62, 593–602. doi: 10.1001/archpsyc.62.6.593, PMID: 15939837

[ref20] Kim J. J. Fanselow M. S. (1992). Modality-specific retrograde amnesia of fear. Science 256, 675–677. doi: 10.1126/science.1585183, PMID: 1585183

[ref21] Krypotos A. M. Effting M. Kindt M. Beckers T. (2015). Avoidance learning: a review of theoretical models and recent developments. Front. Behav. Neurosci. 9:189. doi: 10.3389/fnbeh.2015.00189 26257618 PMC4508580

[ref22] Kwapis J. L. Jarome T. J. Ferrara N. C. Helmstetter F. J. (2017). Updating procedures can reorganize the neural circuit supporting a fear memory. Neuropsychopharmacology 42, 1688–1697. doi: 10.1038/npp.2017.23, PMID: 28139682 PMC5518901

[ref23] Kwapis J. L. Jarome T. J. Lee J. L. Helmstetter F. J. (2015). The retrosplenial cortex is involved in the formation of memory for context and trace fear conditioning. Neurobiol. Learn. Mem. 123, 110–116. doi: 10.1016/j.nlm.2015.06.007, PMID: 26079095 PMC4754129

[ref24] Lee S. H. Choi J. H. Lee N. Lee H. R. Kim J. I. Yu N. K.. (2008). Synaptic protein degradation underlies destabilization of retrieved fear memory. Science 319, 1253–1256. doi: 10.1126/science.1150541, PMID: 18258863

[ref25] MacArdy E. A. Riccio D. C. (1995). Time-dependent changes in the effectiveness of a noncontingent footshock reminder. Learn. Motiv. 26, 29–42. doi: 10.1016/0023-9690(95)90009-8

[ref26] Maren S. Aharonov G. Fanselow M. S. (1997). Neurotoxic lesions of the dorsal hippocampus and Pavlovian fear conditioning in rats. Behav. Brain Res. 88, 261–274. doi: 10.1016/S0166-4328(97)00088-0, PMID: 9404635

[ref27] Maren S. Fanselow M. S. (1995). Synaptic plasticity in the basolateral amygdala induced by hippocampal formation stimulation *in vivo*. J. Neurosci. 15, 7548–7564. doi: 10.1523/JNEUROSCI.15-11-07548.1995, PMID: 7472506 PMC6578043

[ref28] Maren S. Fanselow M. S. (1996). The amygdala and fear conditioning: has the nut been cracked? Neuron 16, 237–240. doi: 10.1016/S0896-6273(00)80041-0, PMID: 8789938

[ref29] McAllister W. R. McAllister D. E. (2006). Recovery of conditioned fear by a single postextinction shock: Effect of similarity of shock contexts and of time following extinction. Learn. Behav. 34, 44–49. doi: 10.3758/BF03192870, PMID: 16786883

[ref30] McIntyre C. K. Miyashita T. Setlow B. Marjon K. D. Steward O. Guzowski J. F.. (2005). Memory-influencing intra-basolateral amygdala drug infusions modulate expression of Arc protein in the hippocampus. Proc. Natl. Acad. Sci. 102, 10718–10723. doi: 10.1073/pnas.0504436102, PMID: 16020527 PMC1175582

[ref31] Nader K. Schafe G. E. Le Doux J. E. (2000). Fear memories require protein synthesis in the amygdala for reconsolidation after retrieval. Nature 406, 722–726. doi: 10.1038/35021052, PMID: 10963596

[ref32] Oleksiak C. R. Ramanathan K. R. Miles O. W. Perry S. J. Maren S. Moscarello J. M. (2021). Ventral hippocampus mediates the context-dependence of two-way signaled avoidance in male rats. Neurobiol. Learn. Mem. 183:107458. doi: 10.1016/j.nlm.2021.107458 34015439 PMC8319050

[ref33] Ortiz S. Latsko M. S. Fouty J. L. Dutta S. Adkins J. M. Jasnow A. M. (2019). Anterior cingulate cortex and ventral hippocampal inputs to the basolateral amygdala selectively control generalized fear. J. Neurosci. 39, 6526–6539. doi: 10.1523/JNEUROSCI.0810-19.2019, PMID: 31209172 PMC6697404

[ref34] Parsons R. G. Gafford G. M. Baruch D. E. Riedner B. A. Helmstetter F. J. (2006). Long-term stability of fear memory depends on the synthesis of protein but not mRNA in the amygdala. Eur. J. Neurosci. 23, 1853–1859. doi: 10.1111/j.1460-9568.2006.04723.x, PMID: 16623842 PMC1698267

[ref35] Pollack G. A. Bezek J. L. Lee S. H. Scarlata M. J. Weingast L. T. Bergstrom H. C. (2018). Cued fear memory generalization increases over time. Learn. Mem. 25, 298–308. doi: 10.1101/lm.047555.118, PMID: 29907637 PMC6004064

[ref36] Poulos A. M. Mehta N. Lu B. Amir D. Livingston B. Santarelli A.. (2016). Conditioning-and time-dependent increases in context fear and generalization. Learn. Mem. 23, 379–385. doi: 10.1101/lm.041400.115, PMID: 27317198 PMC4918784

[ref37] Quinn J. J. Loya F. Ma Q. D. Fanselow M. S. (2005). Dorsal hippocampus NMDA receptors differentially mediate trace and contextual fear conditioning. Hippocampus 15, 665–674. doi: 10.1002/hipo.20088, PMID: 15959918

[ref38] Restivo L. Vetere G. Bontempi B. Ammassari-Teule M. (2009). The formation of recent and remote memory is associated with time-dependent formation of dendritic spines in the hippocampus and anterior cingulate cortex. J. Neurosci. 29, 8206–8214. doi: 10.1523/JNEUROSCI.0966-09.2009, PMID: 19553460 PMC6666032

[ref39] Riccio D. C. Joynes R. L. (2007). Forgetting of stimulus attributes: Some implications for hippocampal models of memory. Learn. Mem. 14, 430–432. doi: 10.1101/lm.617107, PMID: 17554088

[ref40] Roesler R. Parent M. B. LaLumiere R. T. McIntyre C. K. (2021). Amygdala-hippocampal interactions in synaptic plasticity and memory formation. Neurobiol. Learn. Mem. 184:107490. doi: 10.1016/j.nlm.2021.107490 34302951 PMC8435011

[ref41] Rudy J. W. Barrientos R. M. O'reilly R. C. (2002). Hippocampal formation supports conditioning to memory of a context. Behav. Neurosci. 116, 530–538. doi: 10.1037/0735-7044.116.4.530 12148921

[ref42] Rudy J. W. Sutherland R. J. (2008). Is it systems or cellular consolidation? Time will tell. An alternative interpretation of the Morris group’s recent science paper. Neurobiol. Learn. Mem. 89, 366–369. doi: 10.1016/j.nlm.2007.07.017, PMID: 17977757 PMC2471866

[ref43] Salters-Pedneault K. Tull M. T. Roemer L. (2004). The role of avoidance of emotional material in the anxiety disorders. Appl. Prev. Psychol. 11, 95–114. doi: 10.1016/j.appsy.2004.09.001

[ref44] Sara S. J. (2000). Retrieval and reconsolidation: toward a neurobiology of remembering. Learn. Mem. 7, 73–84. doi: 10.1101/lm.7.2.73 10753974

[ref45] Slaker M. L. Harkness J. H. Sorg B. A. (2016). A standardized and automated method of perineuronal net analysis using *Wisteria floribunda* agglutinin staining intensity. IBRO Rep. 1, 54–60. doi: 10.1016/j.ibror.2016.10.001, PMID: 28713865 PMC5507617

[ref46] Sorg B. A. Berretta S. Blacktop J. M. Fawcett J. W. Kitagawa H. Kwok J. C.. (2016). Casting a wide net: role of perineuronal nets in neural plasticity. J. Neurosci. 36, 11459–11468. doi: 10.1523/JNEUROSCI.2351-16.2016, PMID: 27911749 PMC5125213

[ref47] Sugar J. Witter M. P. van Strien N. M. Cappaert N. L. (2011). The retrosplenial cortex: intrinsic connectivity and connections with the (para) hippocampal region in the rat. An interactive connectome. Front. Neuroinform. 5:7. doi: 10.3389/fninf.2011.00007 21847380 PMC3147162

[ref48] Teixeira C. M. Pomedli S. R. Maei H. R. Kee N. Frankland P. W. (2006). Involvement of the anterior cingulate cortex in the expression of remote spatial memory. J. Neurosci. 26, 7555–7564. doi: 10.1523/JNEUROSCI.1068-06.2006 16855083 PMC6674278

[ref49] Todd T. P. Bucci D. J. (2015). Retrosplenial cortex and long-term memory: molecules to behavior. Neural Plast. 2015, 1–9. doi: 10.1155/2015/414173, PMID: 26380115 PMC4562169

[ref50] Todd T. P. Fournier D. I. Bucci D. J. (2019). Retrosplenial cortex and its role in cue-specific learning and memory. Neurosci. Biobehav. Rev. 107, 713–728. doi: 10.1016/j.neubiorev.2019.04.016, PMID: 31055014 PMC6906080

[ref51] Todd T. P. Mehlman M. L. Keene C. S. DeAngeli N. E. Bucci D. J. (2016). Retrosplenial cortex is required for the retrieval of remote memory for auditory cues. Learn. Mem. 23, 278–288. doi: 10.1101/lm.041822.116, PMID: 27194795 PMC4880149

[ref52] Trask S. Ferrara N. C. Grisales K. Helmstetter F. J. (2021b). Optogenetic inhibition of either the anterior or posterior retrosplenial cortex disrupts retrieval of a trace, but not delay, fear memory. Neurobiol. Learn. Mem. 185:107530. doi: 10.1016/j.nlm.2021.107530 34592468 PMC8595712

[ref53] Trask S. Ferrara N. C. Jasnow A. M. Kwapis J. L. (2021c). Contributions of the rodent cingulate-retrosplenial cortical axis to associative learning and memory: A proposed circuit for persistent memory maintenance. Neurosci. Biobehav. Rev. 130, 178–184. doi: 10.1016/j.neubiorev.2021.08.023, PMID: 34450181 PMC8511298

[ref54] Trask S. Helmstetter F. J. (2022). Unique roles for the anterior and posterior retrosplenial cortices in encoding and retrieval of memory for context. Cereb. Cortex 32, 3602–3610. doi: 10.1093/cercor/bhab436, PMID: 35029643 PMC9433420

[ref55] Trask S. Pullins S. E. Ferrara N. C. Helmstetter F. J. (2021a). The anterior retrosplenial cortex encodes event-related information and the posterior retrosplenial cortex encodes context-related information during memory formation. Neuropsychopharmacology 46, 1386–1392. doi: 10.1038/s41386-021-00959-x, PMID: 33580135 PMC8134488

[ref56] Wang D. Fawcett J. (2012). The perineuronal net and the control of CNS plasticity. Cell Tissue Res. 349, 147–160. doi: 10.1007/s00441-012-1375-y 22437874

[ref57] Wiltgen B. J. Silva A. J. (2007). Memory for context becomes less specific with time. Learn. Mem. 14, 313–317. doi: 10.1101/lm.430907, PMID: 17522020

[ref58] Zelikowsky M. Bissiere S. Fanselow M. S. (2012). Contextual fear memories formed in the absence of the dorsal hippocampus decay across time. J. Neurosci. 32, 3393–3397. doi: 10.1523/JNEUROSCI.4339-11.2012, PMID: 22399761 PMC3306617

[ref59] Zhou Y. Riccio D. C. (1996). Manipulation of components of context: The context shift effect and forgetting of stimulus attributes. Learn. Motiv. 27, 400–407. doi: 10.1006/lmot.1996.0023, PMID: 8979939

