# Corrigendum: Generalization and discrimination of inhibitory avoidance differentially engage anterior and posterior retrosplenial subregions

**DOI:** 10.3389/fnbeh.2024.1508609

**Published:** 2024-10-28

**Authors:** Erisa Met Hoxha, Payton K. Robinson, Kaitlyn M. Greer, Sydney Trask

**Affiliations:** ^1^Purdue University Department of Psychological Sciences, West Lafayette, IN, United States; ^2^Purdue University Institute for Integrative Neuroscience, West Lafayette, IN, United States; ^3^Purdue University Center on Aging and the Life Course, West Lafayette, IN, United States

**Keywords:** inhibitory avoidance, generalization, discrimination, retrosplenial cortex, basolateral amygdala

In the published article, there was an error in the Funding statement. R21AG081851 was originally referenced to ST in relation to the publication fee of this article but the publication fee was provided for by the Purdue Open Access Fund. The correct Funding statement appears below.

