# Supplementary material for: Generalization and discrimination of inhibitory avoidance differentially engage anterior and posterior retrosplenial subregions
Source: Front Behav Neurosci. 2024 Jan 17;18:1327858. doi: 10.3389/fnbeh.2024.1327858 (PMC10832059; doi:10.3389/fnbeh.2024.1327858)

Figure 1. Example images (20X objective lens) from the weak shock experiment with zif268 staining in green and DAPI in blue.

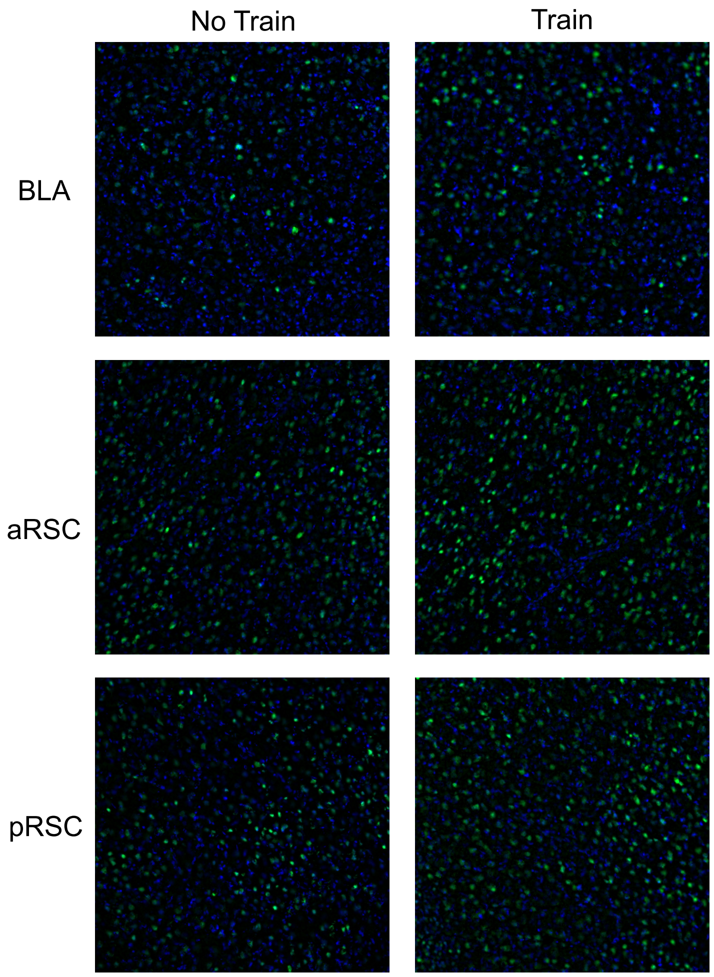

Figure 2. Example images (10X objective lens) from the weak shock experiment with WFA staining in green and DAPI in blue.

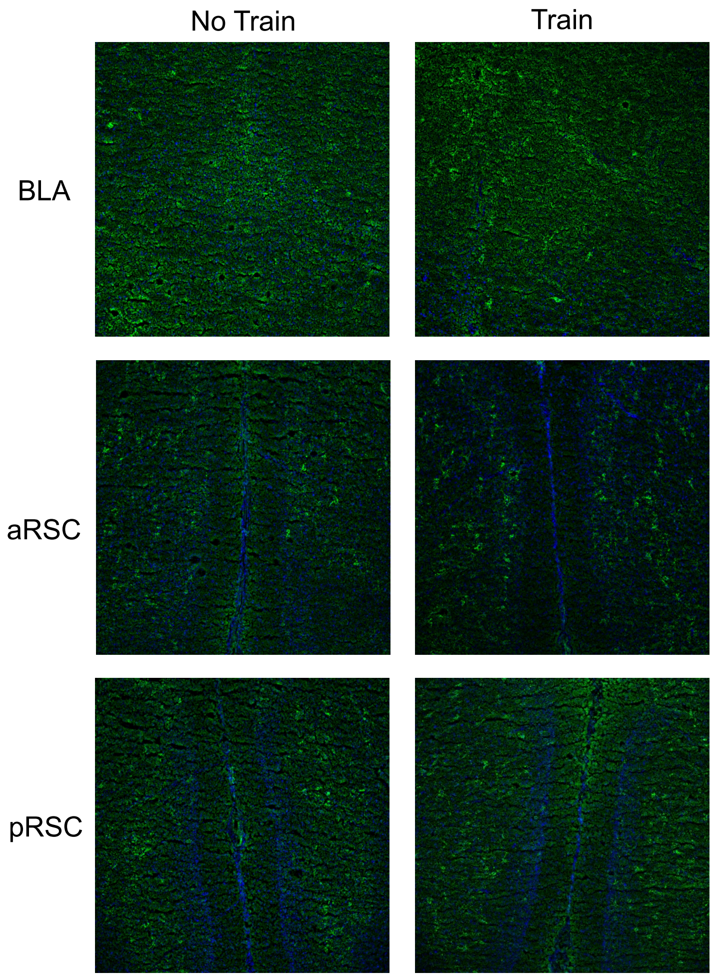

Figure 3. Example images (20X objective lens) from the strong shock experiment with zif268 staining in green and DAPI in blue.

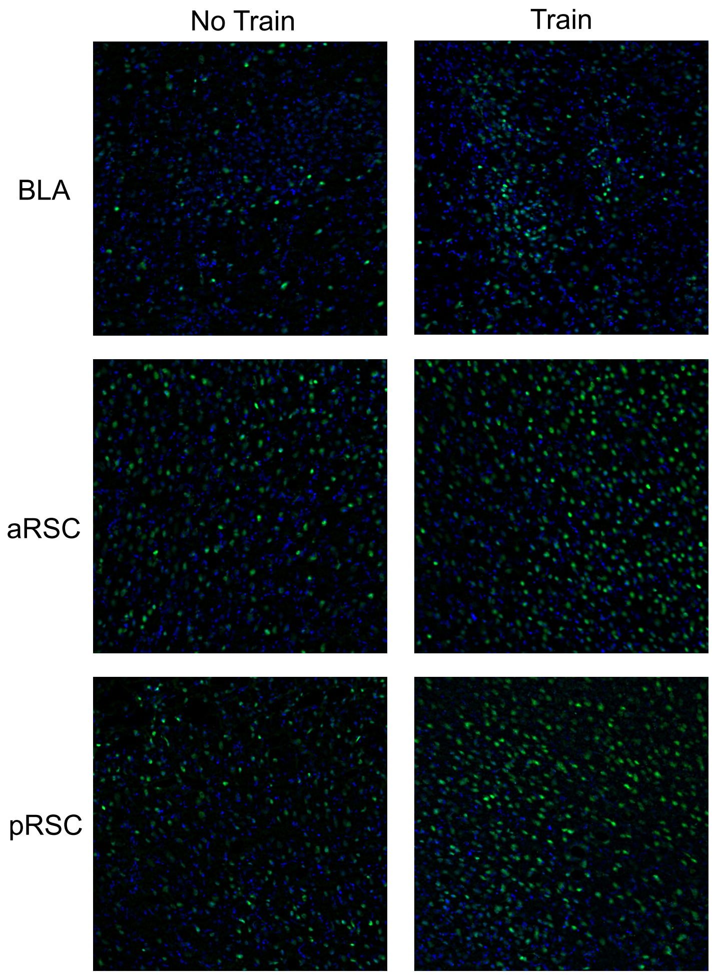

Figure 4. Example images (10X objective lens) from the strong shock experiment with WFA staining in green and DAPI in blue.

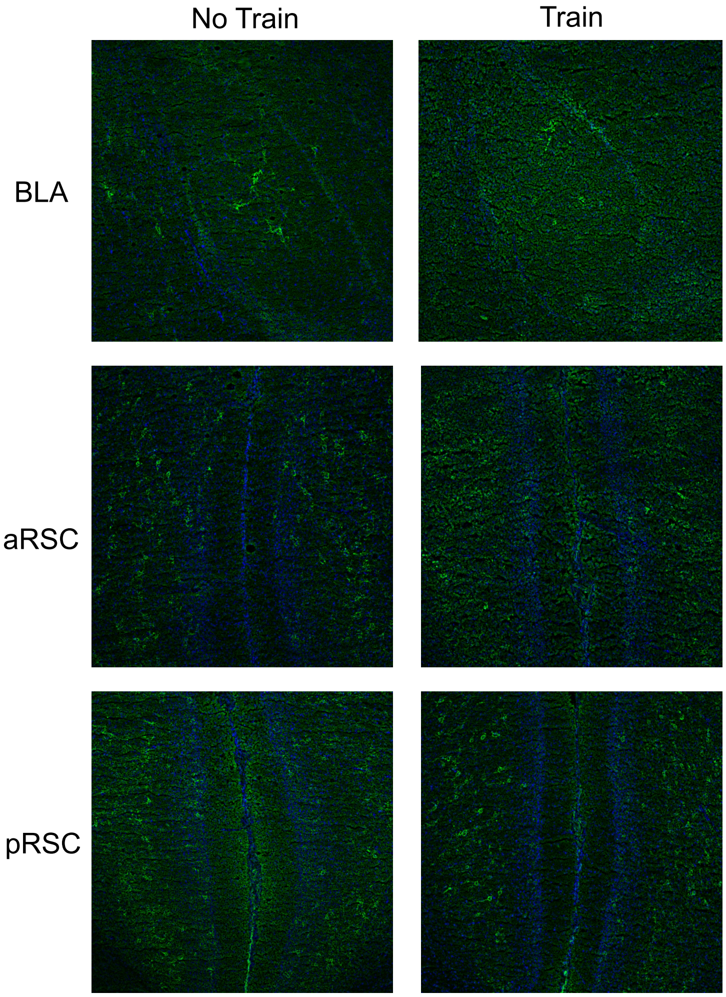

Figure 5. Example images (20X objective lens) from the memory testing experiment with zif268 staining in green and DAPI in blue.

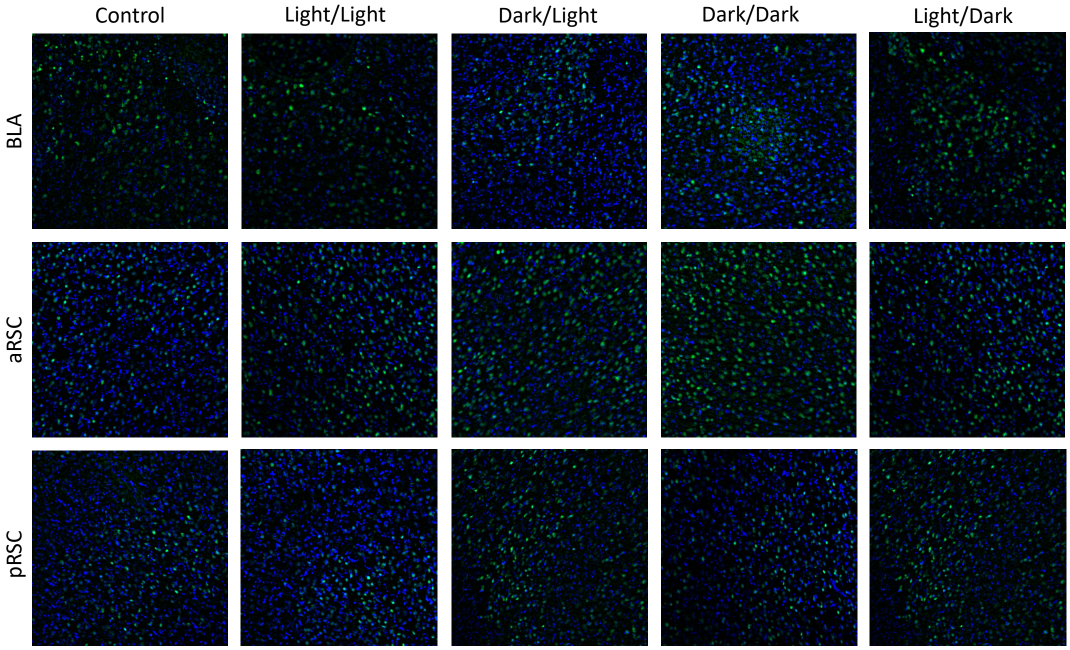

Figure 6. Example images (10X objective lens) from the memory testing experiment with WFA staining in green and DAPI in blue.

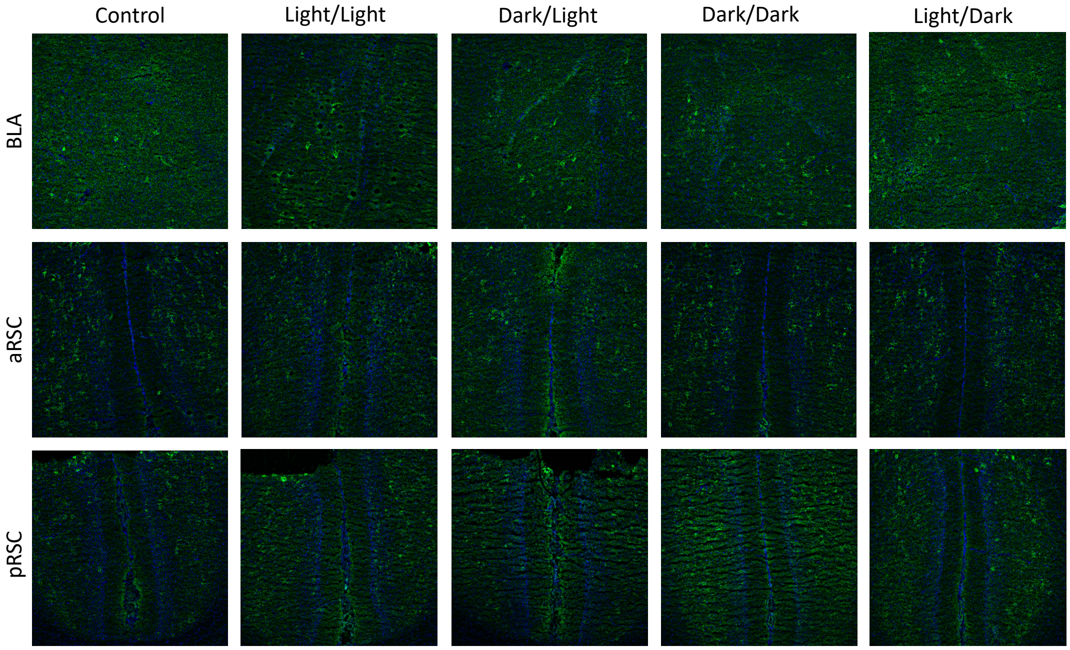

Supplement: Supplementary file 1 [file Data_Sheet_1.PDF]
